# Knowledge, attitudes, and practices toward biologics among systemic lupus erythematosus patients: a cross-sectional study

**DOI:** 10.3389/fpubh.2025.1445576

**Published:** 2025-03-11

**Authors:** Xue Xu, Mengru Du, Peng Lai, Zhiling Zhao, Linyu Geng, Jun Liang, Haifeng Chen, Lingyun Sun

**Affiliations:** ^1^Department of Rheumatology and Immunology, Nanjing Drum Tower Hospital, Affiliated Hospital of Medical School, Nanjing University, Nanjing, Jiangsu, China; ^2^Department of Rheumatology and Immunology, The Affiliated Wuxi People's Hospital of Nanjing Medical University, Wuxi, China

**Keywords:** knowledge, attitude, practice, systemic lupus erythematosus, biologics, cross-sectional study

## Abstract

**Objective:**

This study aimed to investigate the knowledge, attitudes, and practices (KAP) toward biologics among systemic lupus erythematosus (SLE) patients.

**Methods:**

A cross-sectional study was conducted at Nanjing Drum Tower Hospital from March 2023 to January 2024. Demographic information and KAP were obtained through the distribution of self-designed questionnaires.

**Results:**

A total of 543 SLE patients participated in this study, with a mean age of 39.14 ± 13.08 years. The mean scores for knowledge, attitude, and practice were 6.08 ± 5.49 (possible range: 0–32), 33.14 ± 4.01 (possible range: 10–50), and 12.06 ± 3.95 (possible range: 6–30), respectively. Multivariate logistic regression analysis revealed that knowledge score (OR = 1.08, 95% CI: 1.019–1.144, *p* = 0.009), attitude score (OR = 1.476, 95% CI: 1.337–1.63, *p* < 0.001), average monthly income of 5,000–10,000 Yuan (OR = 2.129, 95% CI: 1.327–3.416, *p* = 0.002), and average monthly income of more than 10,000 Yuan (OR = 2.245, 95% CI: 1.184–4.260, *p* = 0.013) were independently associated with proactive practice. Structural equation modeling revealed significant direct effects of knowledge on attitude (*β* = 0.586, *p* < 0.001) and practice (*β* = 0.140, *p* = 0.041). Additionally, attitudes were found to directly influence practice (*β* = 0.628, *p* < 0.001).

**Conclusion:**

SLE patients demonstrated inadequate knowledge, suboptimal attitudes, and passive practices regarding biologics. It is recommended that healthcare providers prioritize education and interventions aimed at enhancing knowledge, fostering positive attitudes, and promoting proactive practices regarding biologic therapies among SLE patients.

## Introduction

Systemic Lupus Erythematosus (SLE), a systemic autoimmune disease, manifests across various organ systems such as the skin, musculoskeletal system, blood vessels, and kidneys (1, 2). Its prevalence varies among populations, affecting approximately 1 in 1000 individuals, with women of childbearing age experiencing a significantly higher incidence rate than men (3). Treatment of SLE involves a range of medications including non-steroidal anti-inflammatory drugs, corticosteroids, anti-malarials, immunosuppressants, and biologic therapies, tailored to address the diverse clinical manifestations and severity of the disease (4, 5). However, patients presenting with refractory disease manifestations, particularly nephritis, often experience severe drug-induced toxicity, which exacerbates organ dysfunction despite the use of traditional therapies. Biologic therapies and therapeutic antibodies have emerged as important therapeutic options in SLE management, not only for refractory cases but also in early stages of disease, with the potential to reduce cumulative damage and modify disease course (6). Despite significant advancements in modern medicine offering various treatment options for SLE, there may be differences in patients’ awareness and understanding of these treatments. Some patients may have knowledge about treatment options and potential side effects, while others may lack essential information. Understanding patients’ knowledge and awareness of treatment is crucial for improving treatment compliance and prognosis.

Within the public health discipline, the interplay between knowledge, attitudes, and behavioral practices is often explored through ‘knowledge, attitude, and practice’ (KAP) surveys, providing a comprehensive framework for understanding how individual behaviors are shaped (7). According to the KAP model, an individual’s practices (behaviors) are influenced by their knowledge and attitudes, highlighting the importance of these elements in guiding health-related decision-making processes (8). Given the recent advancements in biologic treatments for managing severe and refractory cases of SLE, the need to assess patient receptivity to these new therapies has become increasingly critical. This particular patient group offers a unique perspective on the acceptance, potential reservations, and information requirements associated with innovative treatments. Gaining insights into these aspects is vital for developing targeted educational and support strategies, aimed at improving treatment adherence and outcomes, especially in situations where traditional therapies may be inadequate, and the impact on quality of life is significant.

Despite previous KAP studies in this area, there has been a notable gap in research conducted within specific regions (9). Therefore, this study aims to investigate the KAP toward biologics among SLE patients.

## Methods and materials

### Study design and participants

This cross-sectional study was conducted at Nanjing Drum Tower Hospital from March 2023 to January 2024. The study population comprised patients diagnosed with SLE. This study was conducted with the approval of the Institutional Review Board of Nanjing Drum Tower Hospital (2023–639-01) and in accordance with the declaration of Helsinki, and informed consent has been obtained from all participants.

Inclusion criteria: (1) aged 18–70 years old; (2) diagnosed with systemic lupus erythematosus (SLE) according to the 2019 European League against Rheumatism/American College of Rheumatology (EULAR/ACR) classification criteria (10). Exclusion criteria: (1) patients with severe infections, severe liver and kidney dysfunction, unclear consciousness, or pregnancy.

### Questionnaire introduction and quality control

The questionnaire design was guided by the established criteria for the diagnosis and treatment of SLE and recent advancements in biologics for SLE (4, 11), ensuring its content validity and relevance. Prior to implementation, the questionnaire underwent a pilot test with 40 SLE patients from our outpatient clinic who were not included in the final study cohort. The reliability was evaluated using Cronbach’s alpha coefficient, which yielded a value of 0.920, indicating excellent internal consistency.

The final questionnaire, administered in Chinese, comprised four sections: demographic information (gender, age, education level, occupation type, monthly income, etc.), the knowledge dimension, the attitude dimension, and the practice dimension. The knowledge dimension consisted of 10 questions, with the 10th question containing 7 items. Responses were assigned values of 2, 1, and 0 corresponding to “understanding,” “partial understanding, “and “lack of understanding,” respectively, resulting in a score range of 0–32 points. The attitude dimension included 11 questions, with the first 10 questions utilizing a five-point Likert scale ranging from “strongly agree” to “strongly disagree,” scored from 5 to 1. The 11th question did not carry a value, resulting in a score range of 10–50 points. The practice dimension comprised 8 questions, with questions 6 and 8 not being scored. The remaining questions in this section also employed a five-point Likert scale, ranging from “strongly agree/always” (5 points) to “strongly disagree/never” (1 point), resulting in a score range of 6–30 points.

Outpatient or inpatient SLE patients who met the inclusion and exclusion criteria were invited by healthcare providers to complete a questionnaire online after obtaining their consent. If a patient is illiterate or unable to comprehend the questions in the questionnaire, healthcare providers assist by explaining the questions to ensure full comprehension before the patient completes the questionnaire. Each patient completes the questionnaire once, with all questions being mandatory to answer. The questionnaire must be completed in its entirety before submission, ensuring that every question is answered 100%.

### Statistical analysis

Statistical analysis was performed using SPSS 26.0 (IBM Corp., Armonk, N.Y., USA) and AMOS 22.0 (IBM Corp., Armonk, N.Y., USA). Continuous variables were expressed as mean ± standard deviation (SD), and between-group comparisons were conducted using t-tests or analysis of variance (ANOVA). Categorical variables were presented as *n* (%). Univariate and multivariate logistic regression analyses were employed to investigate the risk factors associated with adequate knowledge, positive attitude and proactive practice, which dichotomized using the cut-off value of 70% of the maximum score (12). To address potential confounding factors in the logistic regression analysis, we adjusted for disease severity (using indicators such as frequency of hospitalization and disease stability), treatment accessibility (using income level and medical insurance status), and demographic characteristics (age, gender, education). Variance inflation factors were calculated to check for multicollinearity among these variables. The interactions among the KAP dimensions were evaluated using a structural equation model (SEM). It was hypothesized that knowledge directly affects attitude and practice, while attitude directly affects practice. Two-sided *p*-values <0.05 were considered statistically significant.

The structural equation model was constructed based on the theoretical framework that knowledge influences both attitudes and practices, while attitudes directly affect practices. Model fit was evaluated using multiple indices: chi-square/degree of freedom ratio (CMIN/DF, acceptable if <5), root mean square error of approximation (RMSEA, acceptable if <0.08), incremental fit index (IFI), Tucker-Lewis index (TLI), and comparative fit index (CFI, all acceptable if >0.8). Multicollinearity was assessed using variance inflation factors (VIF), with values <5 considered acceptable. To prevent overfitting, we maintained a minimum ratio of 10 observations per estimated parameter.

## Results

### Demographic characteristics

During the study period, a total of 612 SLE patients were screened for eligibility. Among them, 32 patients were excluded due to severe infections (*n* = 12), severe organ dysfunction (*n* = 8), unclear consciousness (*n* = 5), and pregnancy (*n* = 7). Additionally, 37 patients declined to participate (Supplementary Figure 1). Finally, a total of 543 SLE patients participated in this study, with a mean age of 39.14 ± 13.08 years. Among them, 501 (92.27%) were female, and 236 (43.46%) had obtained a college/bachelor’s degree. Additionally, 188 (34.62%) had received or were receiving biological agent therapy, with 80 (14.73%) having undergone treatment for less than 6 months. Moreover, 387 (71.27%) reported stable remission, and 356 (65.56%) had not experienced repeated hospitalizations due to worsening condition. The mean knowledge, attitude, and practice scores were 6.08 ± 5.49 (possible range: 0–32), 33.14 ± 4.01 (possible range: 10–50), and 12.06 ± 3.95 (possible range: 6–30), respectively. Differences in demographic characteristics revealed that patients with varying education levels, employment types, average monthly incomes, history of receiving biologic therapies, and discontinuation of biological agent therapy were more likely to exhibit diverse knowledge, attitude, and practice scores. Furthermore, variations in the duration of biologic therapy and status of stable remission were associated with differences in knowledge and attitude scores. The reasons for discontinuing biologic therapies and the occurrence of worsening conditions leading to repeated hospitalizations may have contributed to disparities in knowledge scores (*p* < 0.005) (Table 1).

**Table 1:** Baseline characteristics of patients and KAP scores.

	*n* (%)	Knowledge		Attitude		Practice	
		Mean ± SD	*p*	Mean ± SD	*p*	Mean ± SD	*p*
Total	543	6.08 ± 5.49		33.14 ± 4.01		12.06 ± 3.95	
Age (years)	39.14 ± 13.08						
Gender			0.645		0.752		0.158
Male	42 (7.73)	6.45 ± 6.35		32.95 ± 4.10		23.12 ± 4.10	
Female	501 (92.27)	6.05 ± 5.42		33.16 ± 4.00		24.01 ± 3.93	
Education			<0.001		<0.001		<0.001
Elementary school and below	50 (9.21)	3.24 ± 4.04		32.12 ± 3.77		22.24 ± 3.78	
Junior high school	126 (23.20)	4.18 ± 5.04		31.48 ± 3.08		22.50 ± 3.20	
High school/technical school	110 (20.26)	6.05 ± 5.45		32.93 ± 3.36		23.40 ± 4.02	
College/undergraduate	236 (43.46)	7.39 ± 5.56		34.05 ± 4.23		25.04 ± 3.92	
Master’s degree and above	21 (3.87)	9.52 ± 4.04		36.43 ± 5.30		27.24 ± 2.72	
Employment type			<0.001		<0.001		<0.001
Stable long-term employment	232 (42.73)	7.26 ± 5.38		34.15 ± 4.26		25.04 ± 3.87	
Temporary employment	51 (9.39)	5.02 ± 6.16		31.51 ± 3.58		22.25 ± 4.87	
Unemployed	151 (27.81)	5.74 ± 5.89		32.69 ± 3.68		23.14 ± 3.41	
Retired	63 (11.60)	3.51 ± 3.64		31.70 ± 3.33		22.56 ± 3.56	
Student	46 (8.47)	5.93 ± 4.45		33.33 ± 3.69		24.83 ± 3.64	
Average monthly income (CNY)			<0.001		0.009		<0.001
< 5,000	221 (40.70)	5.09 ± 5.25		32.61 ± 3.96		23.08 ± 3.92	
5,000–10,000	212 (39.04)	6.08 ± 5.21		33.19 ± 3.87		24.01 ± 3.63	
10,000–20,000	75 (13.81)	7.67 ± 6.14		33.80 ± 4.17		25.27 ± 4.21	
> 20,000	35 (6.45)	8.91 ± 5.60		34.74 ± 4.26		26.14 ± 3.86	
Duration of illness							
< 6 months	84 (15.47)	4.58 ± 4.73	0.054	32.51 ± 3.55	0.222	24.05 ± 3.94	0.991
6 months −2 years	100 (18.42)	6.32 ± 5.86		33.55 ± 4.45		23.80 ± 3.89	
3–5 years	86 (15.84)	7.07 ± 5.46		33.72 ± 4.32		24.01 ± 4.34	
6–10 years	92 (16.94)	6.15 ± 5.68		32.77 ± 3.34		23.86 ± 3.84	
> 10 years	181 (33.33)	6.13 ± 5.44		33.12 ± 4.08		23.99 ± 3.88	
Have you received or are you currently receiving biologics?			<0.001		<0.001		<0.001
Yes	188 (34.62)	9.95 ± 5.34		35.48 ± 4.36		25.58 ± 3.45	
No	355 (65.38)	4.03 ± 4.34		31.90 ± 3.17		23.08 ± 3.93	
Duration of biologics			<0.001		<0.001		0.092
≤ 6 months	80 (14.73)	8.65 ± 5.16		34.54 ± 3.89		25.31 ± 3.56	
6 months < duration <1 year	43 (7.92)	9.63 ± 4.64		34.67 ± 4.66		25.47 ± 3.35	
1 year ≤ duration ≤2 years	38 (7.00)	12.21 ± 5.07		37.87 ± 3.86		26.79 ± 3.00	
> 2 years	27 (4.97)	11.15 ± 6.19		36.22 ± 4.67		24.85 ± 3.62	
If you have received biologics, have you stopped using it now?			<0.001		0.008		0.021
Yes	47 (8.66)	9.47 ± 5.61		34.02 ± 4.36		24.57 ± 3.68	
No	141 (25.67)	10.11 ± 5.25		35.97 ± 4.26		25.91 ± 3.31	
Reasons for discontinuation			<0.001		0.106		0.086
Symptoms improved or stabilized	10 (1.84)	11.90 ± 5.61		37.1 ± 5.24		26.90 ± 3.31	
Ineffectiveness	11 (2.03)	12.09 ± 7.20		32.36 ± 4.2		23.09 ± 3.30	
Economic reasons	3 (0.55)	8.00 ± 4.58		35.33 ± 4.16		27.00 ± 3.61	
Medication side effects	7 (1.29)	8.71 ± 5.77		33.14 ± 4.14		23.43 ± 3.41	
Other	16 (2.95)	6.75 ± 3.09		33.38 ± 3.36		24.19 ± 3.75	
Is the current condition of systemic lupus erythematosus stable or relieved?			0.028		<0.001		0.780
Yes	387 (71.27)	6.41 ± 5.63		33.52 ± 4.17		23.91 ± 3.93	
No	156 (28.73)	5.26 ± 5.04		32.19 ± 3.39		24.02 ± 4.00	
Have you been repeatedly hospitalized due to worsening of the condition? (exclude regular use of Belimumab)			<0.001		0.080		0.789
Yes, hospitalization frequency per year: ≥ 2 times/year	84 (15.47)	5.85 ± 5.34		32.56 ± 3.55		23.75 ± 3.80	
Yes, hospitalization frequency per year: ≤ 1 time/year	103 (18.97)	7.55 ± 6.30		33.84 ± 4.32		24.15 ± 3.90	
No	356 (65.56)	5.71 ± 5.21		33.07 ± 3.99		23.93 ± 4.01	

### Knowledge, attitudes and practices

Participants’ knowledge distribution revealed that the question garnering the highest “Aware” responses was regarding the lifelong nature of SLE and the necessity for continuous treatment, akin to conditions like hypertension and diabetes (K1), with 39.59% acknowledgment. Conversely, the question with the highest “Partial aware” responses concerned the potential adverse reactions of commonly used lupus drugs (K2), with 55.80% recognition. Conversely, the question receiving the highest “Unaware” responses related to handling adverse reactions when using biologic therapies (K7), with 80.29% unawareness. Regarding understanding of biologic therapies (K10), except for Belimumab (Benlysta), more than 85% of participants were unaware, with only 7.00% aware and 24.86% partially aware of Belimumab (Supplementary Table 1).

Neutral responses dominated attitude dimension items, particularly regarding the side effects of biologic therapies (A6), with 75.14% neutrality, and willingness to use all types of biologic therapies (A11), with over 62.43% neutrality. However, 32.97% expressed agreement with using biologic therapies if covered by medical insurance (A7), while 33.33% agreed on the long-term necessity of biologic therapies in SLE treatment (A9) (Supplementary Table 2).

**Figure 1:**
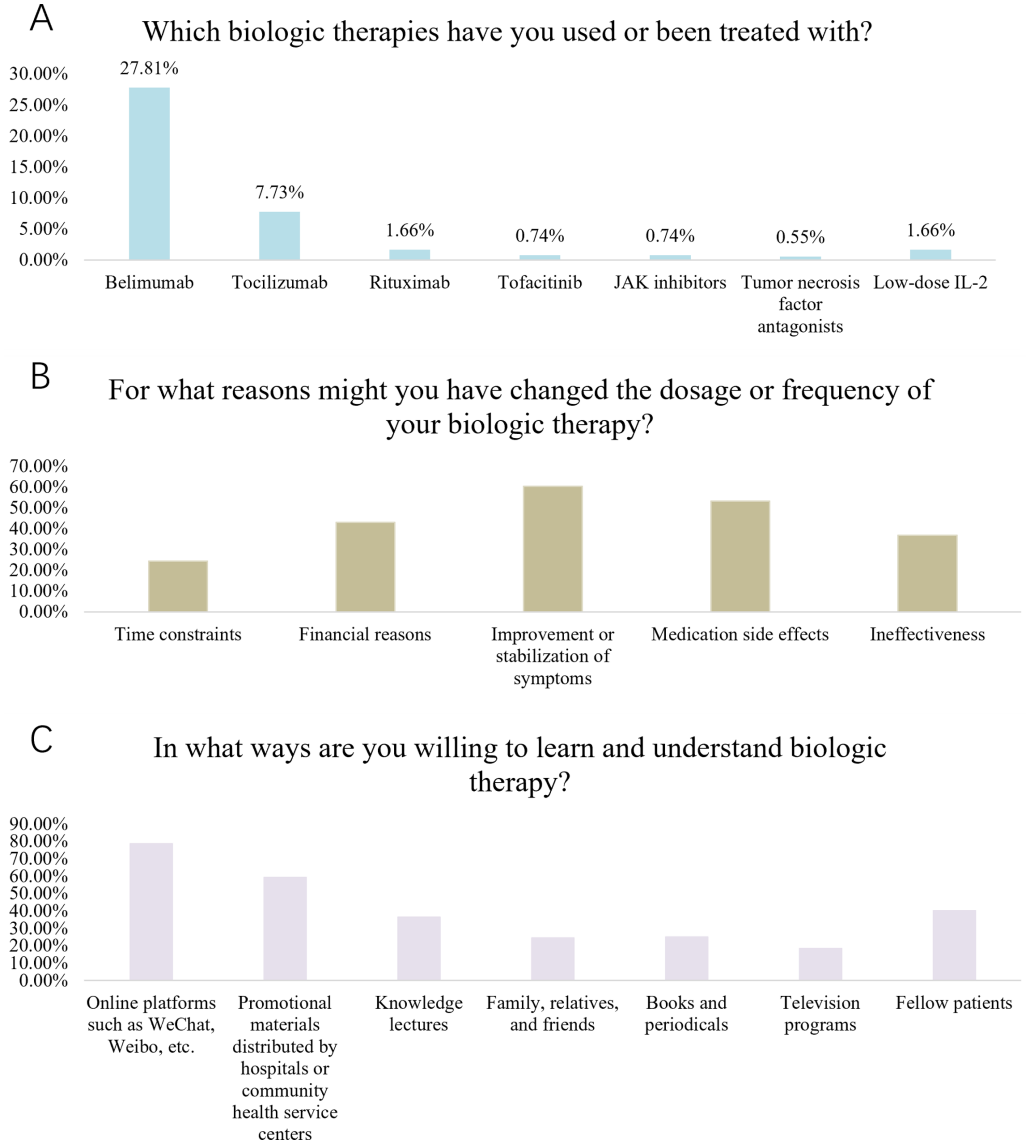
**(A)** biologics Usage Situation. **(B)** Reasons for Changing Dosage and Frequency of biologics. **(C)** Ways of Learning and Understanding biologics.

Patient practice reflected relative positivity, with 28.72% very willing and 50.46% willing to undergo regular reviews during medication to monitor disease progression and medication effects (P4). Similarly, 50.46% expressed willingness to closely monitor medication adverse effects (P3). Notably, 32.97% were neutral regarding recommending effective biologic therapies to other patients (P5) (Supplementary Table 3). Some other more detailed questions also showed that the largest proportion of patients (27.81%) were using or had received treatment with Belimumab (Benlysta) (Figure 1A). Meanwhile, around 60% would have adjusted the dose and frequency of biologic therapies because of improvement or stabilization of their condition (Figure 1B). Moreover, nearly 80% of them were willing to learn about biologic therapies through WeChat, Micro-blog and other online sources (Figure 1C).

### Multivariate logistic regression and SEM results

Multivariate logistic regression showed that high school/technical school education (OR = 2.214, 95% CI: [1.091, 4.49], *p* = 0.028), college education and above (OR = 2.642, 95% CI: [1.301, 5.366], *p* = 0.007), with average monthly income of more than 10,000 Yuan (OR = 2.735, 95% CI: [1.43, 5.23], *p* = 0.002), with duration of illness of 6 months −2 years (OR = 2.319, 95% CI: [1.046, 5.138], *p* = 0.038), had received or currently receiving biologics (OR = 12.354, 95% CI: [7.643, 19.970], *p* < 0.001), stable or relieved current condition of SLE (OR = 1.853, 95% CI: [1.074, 3.196], *p* = 0.027),and with 2 or more times of hospitalization per year (OR = 2.142, 95% CI: [1.081, 4.248], *p* = 0.029) were independently associated with adequate knowledge (Table 2). Meanwhile, knowledge score (OR = 1.137, 95% CI: [1.086, 1.192], *p* < 0.001) and had received or currently receiving biologics (OR = 2.741, 95% CI: [1.729, 4.345], *p* < 0.001) were independently associated with positive attitude (Table 3). Furthermore, knowledge score (OR = 1.08, 95% CI: [1.019, 1.144], *p* = 0.009), attitude score (OR = 1.476, 95% CI: [1.337, 1.63], *p* < 0.001), with average monthly income of 5,000–10,000 Yuan (OR = 2.129, 95% CI: [1.327, 3.416], *p* = 0.002) and with average monthly income of more than 10,000 Yuan (OR = 2.245 95% CI: [1.184, 4.260], *p* = 0.013) were independently associated with proactive practice (Table 4).

**Table 2:** Factors associated with adequate knowledge.

	Univariate analysis		Multivariate analysis	
	OR (95%CI)	*p*	OR (95%CI)	*p*
Age (years)	0.964 (0.950, 0.978)	<0.001	0.984 (0.959, 1.011)	0.250
Gender				
Male	ref.			
Female	0.868 (0.457, 1.651)	0.667		
Education				
Junior high school and below	ref.		ref.	
High school/technical school	2.779 (1.615, 4.783)	<0.001	2.214 (1.091, 4.49)	0.028
College and above	4.535 (2.879, 7.144)	<0.001	2.642 (1.301, 5.366)	0.007
Employment type				
Stable long-term employment	ref.		ref.	
Temporary employment	0.324 (0.161, 0.650)	0.002	0.473 (0.196, 1.144)	0.097
Unemployed	0.476 (0.310, 0.732)	0.001	0.784 (0.408, 1.509)	0.467
Retired	0.274 (0.141, 0.531)	<0.001	0.413 (0.147, 1.158)	0.093
Student	0.677 (0.355, 1.291)	0.236	0.457 (0.183, 1.138)	0.092
Average monthly income (CNY)				
< 5,000	ref.		ref.	
5,000–10,000	1.397 (0.935, 2.088)	0.103	1.387 (0.812, 2.37)	0.231
> 10,000	2.880 (1.793, 4.626)	<0.001	2.735 (1.43, 5.23)	0.002
Duration of illness				
< 6 months	ref.		ref.	
6 months −2 years	2.214 (1.183, 4.143)	0.013	2.319 (1.046, 5.138)	0.038
3–5 years	2.451 (1.286, 4.671)	0.006	1.655 (0.715, 3.832)	0.240
6–10 years	1.171 (0.604, 2.269)	0.641	0.76 (0.329, 1.753)	0.520
> 10 years	1.777 (1.004, 3.146)	0.048	1.21 (0.568, 2.581)	0.621
Have you received or are you currently receiving biologics?				
Yes	12.898 (8.446, 19.697)	<0.001	12.354 (7.643, 19.970)	<0.001
No	ref.		ref.	
Is the current condition of systemic lupus erythematosus stable or relieved?				
Yes	1.566 (1.052, 2.331)	0.027	1.853 (1.074, 3.196)	0.027
No	ref.		ref.	
Have you been repeatedly hospitalized due to worsening of the condition? (exclude regular use of Belimumab)				
Yes, hospitalization frequency per year: ≥ 2 times/year	1.321 (0.811, 2.151)	0.264	2.142 (1.081, 4.248)	0.029
Yes, hospitalization frequency per year: ≤ 1 time/year	1.695 (1.086, 2.645)	0.020	1.757 (0.966, 3.194)	0.065
No	ref.		ref.	

**Table 3:** Factors associated with positive attitude.

	Univariate analysis		Multivariate analysis	
	OR (95%CI)	*p*	OR (95%CI)	*p*
Knowledge score	1.220 (1.169, 1.272)	<0.001	1.137 (1.086, 1.192)	<0.001
Age (years)	0.968 (0.954, 0.982)	<0.001	1.002 (0.979, 1.026)	0.842
Gender				
Male	ref.			
Female	0.951 (0.497, 1.820)	0.880		
Education				
Junior high school and below	ref.		ref.	
High school/technical school	2.064 (1.211, 3.516)	0.008	1.468 (0.773, 2.789)	0.240
College and above	3.558 (2.297, 5.509)	<0.001	1.846 (0.981, 3.471)	0.057
Employment type				
Stable long-term employment	ref.		ref.	
Temporary employment	0.335 (0.167, 0.673)	0.002	0.480 (0.209, 1.103)	0.084
Unemployed	0.508 (0.331, 0.780)	0.002	0.720 (0.405, 1.282)	0.265
Retired	0.256 (0.130, 0.506)	<0.001	0.434 (0.176, 1.073)	0.071
Student	0.701 (0.367, 1.337)	0.28	0.763 (0.341, 1.707)	0.511
Average monthly income (CNY)				
< 5,000	ref.		ref.	
5,000–10,000	1.180 (0.794, 1.755)	0.413	0.911 (0.565, 1.468)	0.701
> 10,000	1.855 (1.162, 2.963)	0.010	0.908 (0.504, 1.636)	0.748
Duration of illness				
< 6 months	ref.			
6 months −2 years	1.529 (0.834, 2.803)	0.170		
3–5 years	1.671 (0.894, 3.123)	0.107		
6–10 years	0.877 (0.462, 1.665)	0.688		
> 10 years	1.241 (0.717, 2.147)	0.441		
Have you received or are you currently receiving biologics?				
Yes	5.524 (3.761, 8.114)	<0.001	2.741 (1.729, 4.345)	<0.001
No	ref.		ref.	
Is the current condition of systemic lupus erythematosus stable or relieved?				
Yes	1.666 (1.115, 2.489)	0.013	1.523 (0.956, 2.426)	0.077
No	ref.		ref.	
Have you been repeatedly hospitalized due to worsening of the condition? (exclude regular use of Belimumab)				
Yes, hospitalization frequency per year: ≥ 2 times/year	0.779 (0.468, 1.298)	0.338		
Yes, hospitalization frequency per year: ≤ 1 time/year	1.349 (0.864, 2.105)	0.188		
No	ref.			

**Table 4:** Factors associated with proactive practice.

	Univariate analysis		Multivariate analysis	
	OR (95%CI)	*p*	OR (95%CI)	*p*
Knowledge score	1.195 (1.140, 1.253)	<0.001	1.08 (1.019, 1.144)	0.009
Attitude score	1.533 (1.401, 1.677)	<0.001	1.476 (1.337, 1.63)	<0.001
Age (years)	0.977 (0.964, 0.990)	0.001	1.014 (0.99, 1.039)	0.243
Gender				
Male	ref.			
Female	1.315 (0.695, 2.487)	0.401		
Education				
Junior high school and below	ref.		ref.	
High school/technical school	1.403 (0.868, 2.266)	0.167	0.771 (0.421, 1.414)	0.401
College and above	3.223 (2.139, 4.856)	<0.001	1.363 (0.730, 2.547)	0.331
Employment type				
Stable long-term employment	ref.		ref.	
Temporary employment	0.366 (0.197, 0.681)	0.001	0.841 (0.394, 1.798)	0.656
Unemployed	0.491 (0.319, 0.755)	0.001	0.868 (0.476, 1.583)	0.644
Retired	0.393 (0.222, 0.697)	0.001	0.766 (0.327, 1.796)	0.540
Student	0.967 (0.479, 1.954)	0.926	1.836 (0.741, 4.551)	0.189
Average monthly income (CNY)				
< 5,000	ref.		ref.	
5,000–10,000	2.192 (1.481, 3.246)	<0.001	2.129 (1.327, 3.416)	0.002
> 10,000	3.551 (2.103, 5.995)	<0.001	2.245 (1.184, 4.260)	0.013
Duration of illness				
< 6 months	ref.			
6 months −2 years	0.691 (0.373, 1.282)	0.242		
3–5 years	0.681 (0.359, 1.289)	0.237		
6–10 years	0.690 (0.368, 1.295)	0.248		
> 10 years	0.688 (0.395, 1.199)	0.187		
Have you received or are you currently receiving biologics?				
Yes	2.909 (1.941, 4.358)	<0.001	0.879 (0.502, 1.537)	0.650
No	ref.		ref.	
Is the current condition of systemic lupus erythematosus stable or relieved?				
Yes	1.146 (0.781, 1.680)	0.486		
No	ref.			
Have you been repeatedly hospitalized due to worsening of the condition? (exclude regular use of Belimumab)				
Yes, hospitalization frequency per year: ≥ 2 times/year	0.889 (0.546, 1.448)	0.637		
Yes, hospitalization frequency per year: ≤ 1 time/year	1.026 (0.650, 1.620)	0.912		
No	ref.			

**Figure 2:**
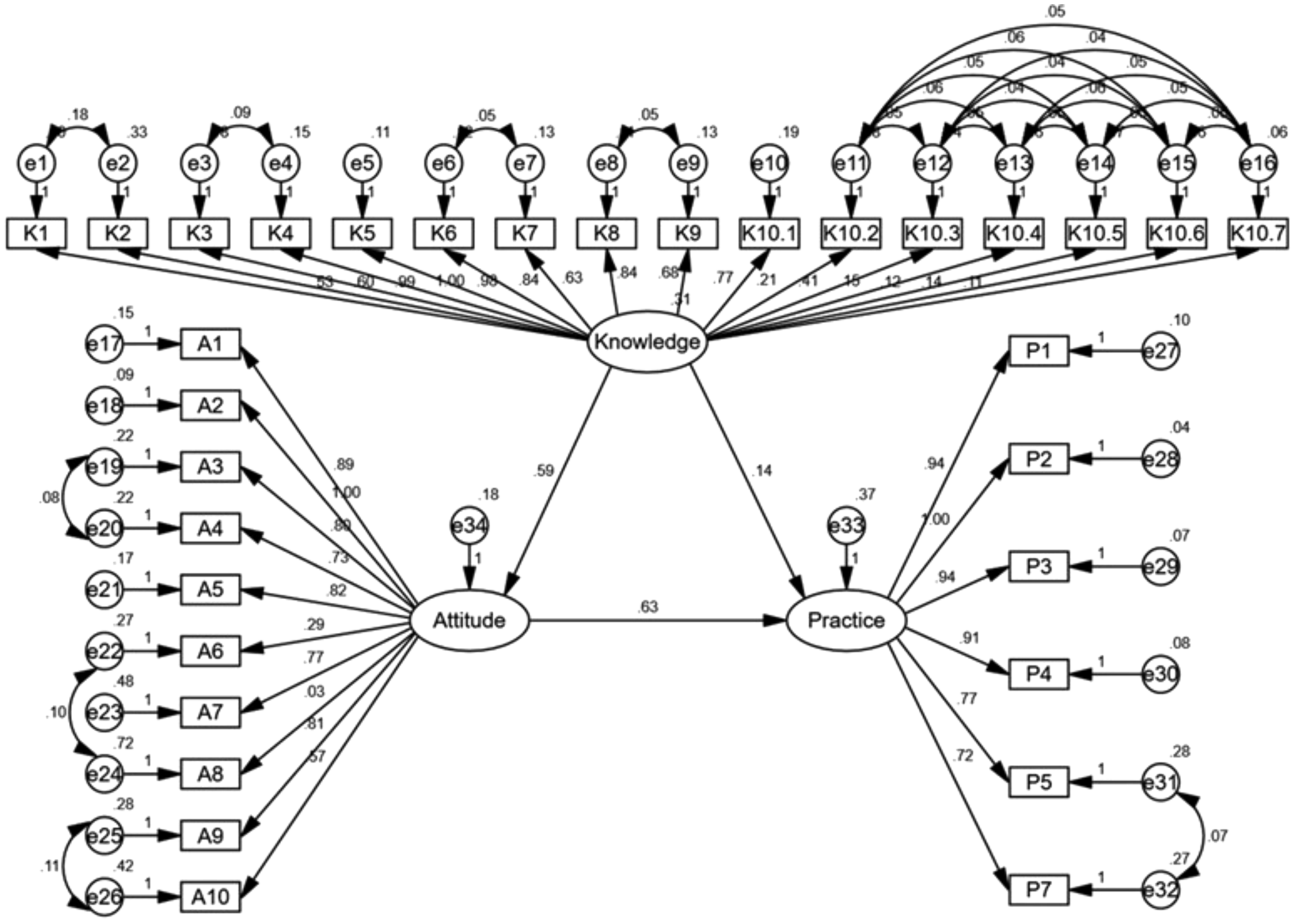
SEM Model.

The SEM demonstrate a highly favorable model fit indices, suggesting an excellent-fitting model (Supplementary Table 4), and shown that knowledge had direct effects on attitude (*β* = 0.586, *p* < 0.001) and practice (*β* = 0.140, *p* = 0.041). Moreover, attitudes have a direct impact on practice (*β* = 0.628, *p* < 0.001) (Table 5; Figure 2).

**Table 5:** SEM results.

	Estimate	*p*
Attitude < Knowledge	0.586	<0.001
Practice < Attitude	0.628	<0.001
Practice < Knowledge	0.140	0.041

## Discussion

SLE patients demonstrated insufficient knowledge, suboptimal attitudes, and inactive practices regarding biologics. Consequently, it is imperative to implement targeted educational interventions to enhance patient understanding and promote proactive engagement with biologic treatments.

Similar to findings from another study, patients with SLE exhibited inadequate knowledge, suboptimal attitudes, and inactive practices concerning biologics (9). This observation highlights potential deficiencies in patient education and emphasizes the necessity for interventions aimed at enhancing patient comprehension and involvement with biologic therapies.

Our multivariate analysis identified education level as a significant predictor of knowledge about biologics. Participants with higher education levels demonstrated better understanding of biologic therapies, consistent with previous studies showing that educational background influences comprehension of complex medical information in SLE management. Similarly, employment status and income levels were associated with higher KAP scores, suggesting that socioeconomic factors play a crucial role in patients’ engagement with biologic therapies (13). The strong association between biologics use and knowledge scores remained significant after adjusting for potential confounders including disease severity and treatment accessibility. This robust relationship suggests that direct experience with biologics substantially influences patients’ understanding, independent of other factors. However, we acknowledge that unmeasured confounders such as healthcare access patterns and physician preferences may still affect this relationship. Similarly, employment status and income levels demonstrated significant associations with KAP scores, reflecting socioeconomic disparities in health literacy and access to healthcare resources (14, 15).

Furthermore, the study highlights the impact of biologic treatment on patient KAP outcomes. Participants receiving biologics reported significantly higher scores across all three domains, indicating the pivotal role of ongoing medical intervention in improving patient education, perceptions, and behaviors. This finding corroborates existing evidence suggesting that effective disease management strategies, including biologic treatments, can positively influence patient engagement and health outcomes (16, 17). Additionally, the duration of biologic treatment emerged as a significant predictor of better KAP scores, emphasizing the importance of sustained therapeutic interventions in promoting patient education and empowerment.

The SEM analysis corroborated the interplay between knowledge, attitudes, and practices in shaping patient behaviors regarding biologic therapies. The SEM analysis demonstrated that knowledge had direct effects on attitude and practice, while attitudes had a direct impact on practice. These findings align well with the core tenets of the Theory of Planned Behavior, which posits that individuals’ intentions to engage in a behavior are influenced by their attitudes toward the behavior, subjective norms (perceived social pressure to perform or not perform the behavior), and perceived behavioral control (perceived ease or difficulty of performing the behavior) (18, 19).

The study highlights a notable discrepancy in knowledge levels among participants regarding SLE and its treatment modalities. While awareness of the chronic nature of SLE and the importance of continuous treatment was relatively high, understanding of specific biological agents and their potential adverse effects appeared limited. Additionally, interactive educational sessions led by healthcare professionals can offer opportunities for patients to ask questions, clarify misconceptions, and deepen their understanding of treatment options. Establishing support groups or online forums where patients can share experiences and exchange information may also enhance knowledge dissemination and empower patients to make informed decisions about their care (20).

The study reveals a spectrum of attitudes among participants regarding the safety, efficacy, and financial implications of biologic therapies for SLE. Many individuals recognize the potential therapeutic benefits of these treatments in managing disease activity and reducing relapses. This observation aligns with the treatment recommendations for SLE, as patients in this study demonstrate a strong understanding of the advantages of biologic therapies (6). However, concerns regarding financial strain and potential side effects are widespread. To foster positive attitudes and improve treatment acceptance, healthcare providers must engage in open and transparent communication with patients. Shared decision-making processes, guided by comprehensive information about treatment options, risks, and benefits, can help address patient concerns and align treatment decisions with individual preferences and priorities. Moreover, access to financial assistance programs and resources is essential to alleviate the financial burden associated with biologic therapies for patients with limited resources or inadequate insurance coverage. By addressing patient-specific concerns and providing support throughout the treatment journey, healthcare providers can promote trust, collaboration, and mutual respect in the patient-provider relationship (20, 21).

The findings underscore the importance of patient engagement in self-management practices and adherence to treatment protocols during biologic therapy for SLE. While the majority of participants express willingness to adhere to prescribed regimens and undergo regular monitoring, opportunities for active learning and self-monitoring appear underutilized. Our findings through structural equation modeling revealed a clear pathway where knowledge influenced attitudes, which in turn affected practices regarding biologic therapies. This suggests that improving patients’ understanding of biologics could lead to more positive attitudes and ultimately better treatment practices. Notably, patients who had received biologics showed more favorable attitudes and practices, indicating that direct experience with these treatments may enhance treatment acceptance and compliance. These approaches align with the evolving landscape of healthcare delivery, which increasingly emphasizes the integration of technology and patient engagement to optimize outcomes (22). Encouraging patient participation in support groups and educational workshops can also foster peer support, enhance self-efficacy, and equip patients with practical skills for managing SLE and biologic therapies effectively. By prioritizing patient-centered care and fostering collaborative partnerships between patients and providers, healthcare teams can optimize treatment outcomes and improve quality of life for individuals living with SLE (23, 24).

This study has several limitations. Firstly, this single-center study was conducted at a tertiary hospital in eastern China, which may limit the generalizability of our findings. When comparing our study population with previous multi-center studies in China, while our cohort showed comparable gender distribution and age range to national data, we noted some differences. Our participants had relatively higher education and income levels, likely reflecting the urban setting of our hospital. Additionally, our biological agent usage rate was higher than typically reported in national studies, possibly due to our hospital’s tertiary care status. These differences should be considered when interpreting our findings. Secondly, reliance on self-reported data through questionnaires may introduce response biases or inaccuracies, potentially impacting the validity of the results. Thirdly, the cross-sectional design precludes establishing causal relationships between variables and only provides a snapshot of the KAP status at a specific point in time. However, despite these limitations, this study offers valuable insights into SLE patients’ KAP toward biologics. Its large sample size, robust statistical analyses including multivariate logistic regression and structural equation modeling, and comprehensive assessment of KAP strengthen the validity and reliability of the findings. Additionally, identification of factors associated with KAP can inform targeted interventions to improve patient understanding and management of biologic therapies in SLE.

In conclusion, SLE patients demonstrated insufficient knowledge, suboptimal attitudes, and inactive practices regarding biologic therapies. Based on our findings, we recommend that healthcare providers: (1) implement systematic education programs about biologic therapies, particularly targeting patients with lower education levels; (2) address financial concerns through early discussion of insurance coverage and available assistance programs; and (3) utilize experienced patients’ positive outcomes to help educate new patients about biologic therapies.

## Data Availability

The datasets presented in this study can be found in online repositories. The names of the repository/repositories and accession number(s) can be found in the article/Supplementary material.

## References

[1] Knight JS Mazza LF Yalavarthi S Sule G Ali RA Hodgin JB. Ectonucleotidase-mediated suppression of lupus autoimmunity and vascular dysfunction. Front Immunol. (2018) 9:1322. doi: 10.3389/fimmu.2018.01322, PMID: 29942314 PMC6004379

[2] Prabakaran T Troldborg A Kumpunya S Alee I Marinković E Windross SJ. A STING antagonist modulating the interaction with STIM1 blocks ER-to-Golgi trafficking and inhibits lupus pathology. EBioMedicine. (2021) 66:103314. doi: 10.1016/j.ebiom.2021.103314, PMID: 33813142 PMC8047499

[3] Kang Y Zhang ZJ Zhao XY Zhang ZQ Sheng PY Liao WM. Total hip arthroplasty for vascular necrosis of the femoral head in patients with systemic lupus erythematosus: a midterm follow-up study of 28 hips in 24 patients. Eur J Orthop Surg Traumatol. (2013) 23:73–9. doi: 10.1007/s00590-012-0939-6, PMID: 23412411

[4] Fanouriakis A Kostopoulou M Alunno A Aringer M Bajema I Boletis JN. 2019 update of the EULAR recommendations for the management of systemic lupus erythematosus. Ann Rheum Dis. (2019) 78:736–45. doi: 10.1136/annrheumdis-2019-215089, PMID: 30926722

[5] Moussa T Abdelhak M Edens C. Pseudotumor cerebri syndrome in children with systemic lupus erythematosus: case series and review. Pediatr Rheumatol Online J. (2022) 20:29. doi: 10.1186/s12969-022-00688-5, PMID: 35428311 PMC9013147

[6] Fanouriakis A Kostopoulou M Andersen J Aringer M Arnaud L Bae S-C. EULAR recommendations for the management of systemic lupus erythematosus: 2023 update. Ann Rheum Dis. (2024) 83:15–29. doi: 10.1136/ard-2023-224762, PMID: 37827694

[7] Aerts C Revilla M Duval L Paaijmans K Chandrabose J Cox H. Understanding the role of disease knowledge and risk perception in shaping preventive behavior for selected vector-borne diseases in Guyana. PLoS Negl Trop Dis. (2020) 14:e0008149. doi: 10.1371/journal.pntd.0008149, PMID: 32251455 PMC7170267

[8] Alzghoul BI Abdullah NA. Pain management practices by nurses: an application of the knowledge, attitude and practices (KAP) model. Global J Health Sci. (2015) 8:154–60. doi: 10.5539/gjhs.v8n6p154, PMID: 26755474 PMC4954874

[9] Liu X Song Y Wan L Du R. Knowledge, attitudes, and practices among patients with systemic lupus erythematosus toward disease management and biologic therapy. J Multidiscip Healthc. (2024) 17:937–47. doi: 10.2147/jmdh.S444619, PMID: 38455274 PMC10918590

[10] Aringer M Costenbader K Daikh D Brinks R Mosca M Ramsey-Goldman R. 2019 European league against rheumatism/American College of Rheumatology classification criteria for systemic lupus erythematosus. Ann Rheum Dis. (2019) 78:1151–9. doi: 10.1136/annrheumdis-2018-214819, PMID: 31383717

[11] Chan J Walters GD Puri P Jiang SH. Safety and efficacy of biological agents in the treatment of systemic lupus erythematosus (SLE). BMC rheumatology. (2023) 7:37. doi: 10.1186/s41927-023-00358-3, PMID: 37807057 PMC10561476

[12] Lee F Suryohusodo AA. Knowledge, attitude, and practice assessment toward COVID-19 among communities in East Nusa Tenggara, Indonesia: a cross-sectional study. Front Public Health. (2022) 10:957630. doi: 10.3389/fpubh.2022.957630, PMID: 36388283 PMC9659730

[13] Katz P Dall'Era M Trupin L Rush S Murphy LB Lanata C. Impact of limited health literacy on patient-reported outcomes in systemic lupus erythematosus. Arthritis Care Res. (2021) 73:110–9. doi: 10.1002/acr.24361, PMID: 32741118 PMC7775267

[14] Maheswaranathan M Cantrell S Eudy AM Rogers JL Clowse MEB Hastings SN. Investigating health literacy in systemic lupus erythematosus: a descriptive review. Curr Allergy Asthma Rep. (2020) 20:79. doi: 10.1007/s11882-020-00978-6, PMID: 33184709 PMC8261622

[15] Shenavandeh S Mani A Eazadnegahdar M Nekooeian A. Medication adherence of patients with systemic lupus erythematosus and rheumatoid arthritis considering the psychosocial factors, health literacy and current life concerns of patients. Curr Rheumatol Rev. (2021) 17:412–20. doi: 10.2174/1573397117666210301144651, PMID: 33645485

[16] Lazar S Kahlenberg JM. Systemic lupus erythematosus: new diagnostic and therapeutic approaches. Annu Rev Med. (2023) 74:339–52. doi: 10.1146/annurev-med-043021-032611, PMID: 35804480

[17] Wang Y Huang Z Xiao Y Wan W Yang X. The shared biomarkers and pathways of systemic lupus erythematosus and metabolic syndrome analyzed by bioinformatics combining machine learning algorithm and single-cell sequencing analysis. Front Immunol. (2022) 13:1015882. doi: 10.3389/fimmu.2022.1015882, PMID: 36341378 PMC9627509

[18] Kariburyo F Xie L Sah J Li N Lofland JH. Real-world medication use and economic outcomes in incident systemic lupus erythematosus patients in the United States. J Med Econ. (2020) 23:1–9. doi: 10.1080/13696998.2019.1678170, PMID: 31589081

[19] Keeling SO Alabdurubalnabi Z Avina-Zubieta A Barr S Bergeron L Bernatsky S. Canadian rheumatology association recommendations for the assessment and monitoring of systemic lupus erythematosus. J Rheumatol. (2018) 45:1426–39. doi: 10.3899/jrheum.171459, PMID: 30173152

[20] Jatwani K Chugh K Osholowu OS Jatwani S. Tumid lupus erythematosus and systemic lupus erythematosus: a report on their rare coexistence. Cureus. (2020) 12:e7545. doi: 10.7759/cureus.7545, PMID: 32377493 PMC7199909

[21] Al-Khaldi M Alsabbagh M. Systemic lupus erythematosus presented with bilateral orbital edema and negative serology. Case Rep Rheumatol. (2019) 2019:7140534–5. doi: 10.1155/2019/7140534, PMID: 31662937 PMC6778949

[22] Kuwabara A Su S Krauss J. Utilizing digital health Technologies for Patient Education in lifestyle medicine. Am J Lifestyle Med. (2020) 14:137–42. doi: 10.1177/1559827619892547, PMID: 32231478 PMC7092400

[23] Greiling TM Dehner C Chen X Hughes K Iñiguez AJ Boccitto M. Commensal orthologs of the human autoantigen Ro60 as triggers of autoimmunity in lupus. Sci Transl Med. (2018) 10:10. doi: 10.1126/scitranslmed.aan2306, PMID: 29593104 PMC5918293

[24] Song Y Wei F Liu Y Han F Ma L Zhuang Y. IL-33/ST2 activation is involved in Ro60-regulated photosensitivity in cutaneous lupus erythematosus. Mediat Inflamm. (2022) 2022:4955761–15. doi: 10.1155/2022/4955761, PMID: 35909659 PMC9328989

