# Supplementary figures and images for: Knowledge, attitudes, and practices toward biologics among systemic lupus erythematosus patients: a cross-sectional study
Source: Front Public Health. 2025 Mar 11;13:1445576. doi: 10.3389/fpubh.2025.1445576 (PMC11933085; doi:10.3389/fpubh.2025.1445576)

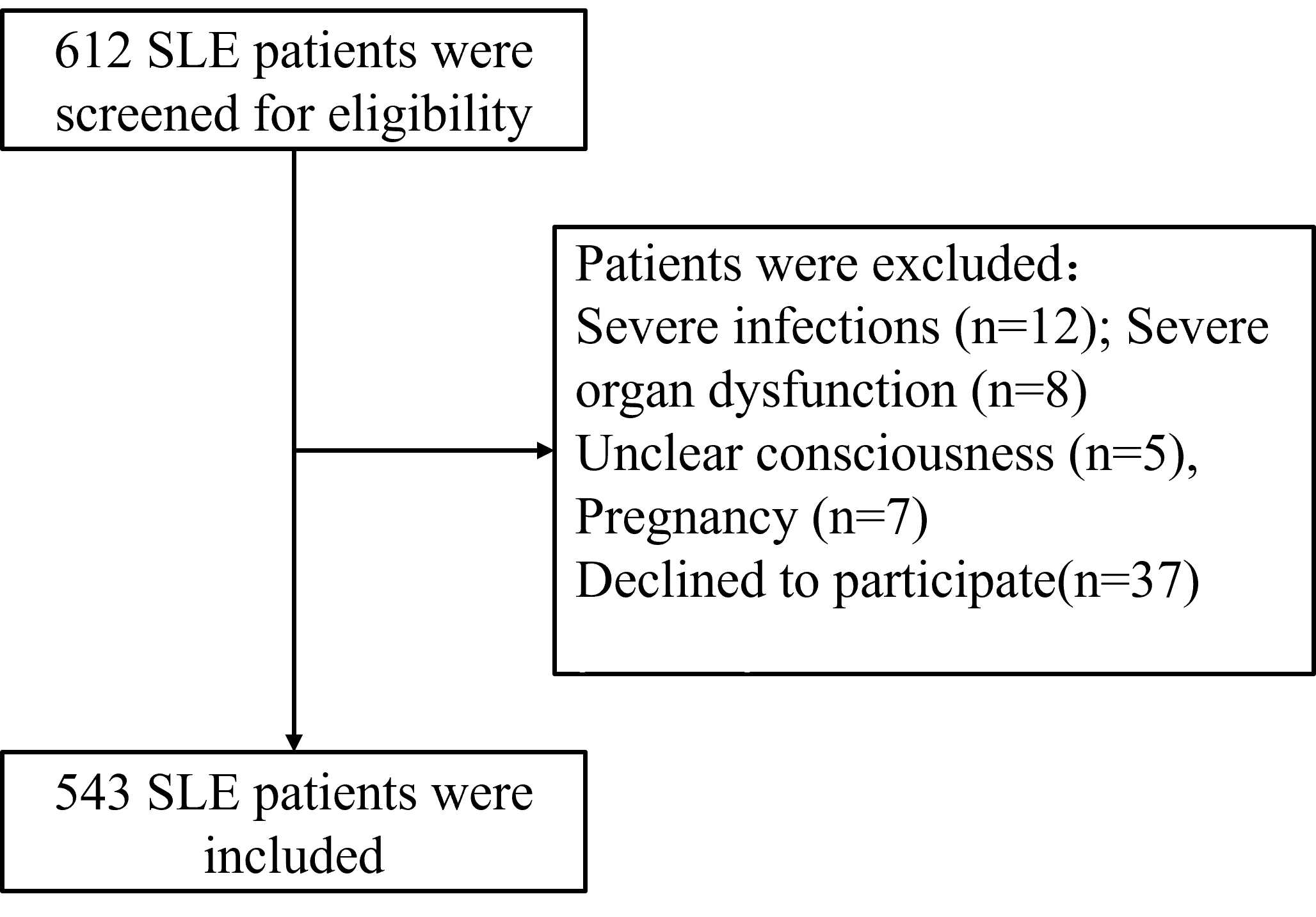

Supplement: Supplementary file 1 [file Image_1.tif]
