# Supplementary material for: Knowledge, attitudes, and practices toward biologics among systemic lupus erythematosus patients: a cross-sectional study
Source: Front Public Health. 2025 Mar 11;13:1445576. doi: 10.3389/fpubh.2025.1445576 (PMC11933085; doi:10.3389/fpubh.2025.1445576)
Supplement: Supplementary file 2 [file Table_1.docx]

**Supplementary table 1 Responses for Each Item in the Knowledge Dimension**

| Items, n (%) | Aware | Partial aware | Unaware |
| --- | --- | --- | --- |
| 1. Are you aware that systemic lupus erythematosus is a lifelong disease, similar to hypertension and diabetes, requiring long-term treatment and should not be abruptly discontinued? | 215(39.59) | 246( 45.30) | 82(15.10) |
| 2. Are you aware of the potential adverse reactions of commonly used drugs in the lupus treatment field, such as obesity and hyperglycemia caused by steroids, amenorrhea caused by cyclophosphamide, liver and kidney function damage caused by tacrolimus, and increased risk of opportunistic infections caused by mycophenolate mofetil? | 112(20.63) | 303(55.80) | 128(23.57) |
| 3. Are you aware that systemic lupus erythematosus can be treated with biologics? | 75(13.81) | 228(41.99) | 240(44.20) |
| 4. Are you aware that the use of biologic therapies can significantly increase the complete and partial remission rates of patients, reduce disease activity, disease recurrence rates, and decrease the dosage of steroids? | 63(11.60) | 218(40.15) | 262(48.25) |
| 5. Are you aware of the duration, dosage, and administration methods of biologic therapies? | 45(8.29) | 150(27.62) | 348(64.09) |
| 6. Are you aware of the potential adverse reactions such as infection and tumors during biologics? | 32(5.89) | 128(23.57) | 383(70.53) |
| 7. Are you aware of how to handle adverse reactions when using biologic therapies? | 21(3.87) | 86(15.84) | 436(80.29) |
| 8. Are you aware that early use of belimumab and tocilizumab can improve the prognosis of lupus patients? | 28(5.16) | 134(24.68) | 381(70.17) |
| 9. Are you aware that if one type of biologic agent is ineffective in treating lupus (e.g., belimumab), another biologic agent (e.g., tocilizumab) can be used? | 19(3.50) | 117(21.55) | 407(74.95) |
| 10. Level of understanding of the following biologic therapies (All to be answered): |  |  |  |
| Belimumab (Benlysta) | 38(7.00) | 135(24.86) | 370(68.14) |
| Rituximab (Rituxan) | 7(1.29) | 28(5.16) | 508(93.55) |
| Tocilizumab (Actemra) | 14(2.58) | 67(12.34) | 462(85.08) |
| Tofacitinib (Xeljanz) | 4(0.74) | 24(4.42) | 515(94.84) |
| JAK inhibitors (Tofacitinib, Baricitinib, Ruxolitinib, Upadacitinib, etc.) | 4(0.74) | 24(4.42) | 515(94.84) |
| Tumor necrosis factor antagonists (Adalimumab, Etanercept, Infliximab, etc.) | 5(0.92) | 20(3.68) | 518(95.40) |
| Low-dose IL-2 | 3(0.55) | 22(4.05) | 518(95.40) |

**Supplementary table 2 Responses for Each Item in the Attitude Dimension**

| Items, n (%) | Strongly agree | Agree | Neutral | Disagree | Strongly disagree |
| --- | --- | --- | --- | --- | --- |
| 1. I believe that injecting biologic therapies is safer and more effective than taking other oral medications. P | 22(4.05) | 131(24.13) | 368(67.77) | 20(3.68) | 2(0.37) |
| 2. I believe that biologic therapies can better control systemic lupus erythematosus and its complications, reducing relapses. P | 27(4.97) | 157(28.91) | 349(64.27) | 9(1.66) | 1(0.18) |
| 3. I believe that biologic therapies have reduced my use of steroids, or I have stopped using steroids. P | 19(3.50) | 129(23.76) | 358(65.93) | 34(6.26) | 3(0.55) |
| 4. I believe that biologic therapies have reduced my dosage of immunosuppressants. P | 16(2.95) | 119(21.92) | 379(69.80) | 24(4.42) | 5(0.92) |
| 5. I believe that biologic therapies should be used early, rather than waiting for other medications to be ineffective. P | 21(3.87) | 138(25.41) | 366(67.40) | 16(2.95) | 2(0.37) |
| 6. I believe that biologic therapies have significant side effects, and I am unwilling to use them. N | 1(0.18) | 31(5.71) | 408(75.14) | 92(16.94) | 11(2.03) |
| 7. I believe that using biologic therapies will increase my financial burden, but I am willing to use them if they are covered by medical insurance. P | 90(16.57) | 179(32.97) | 257(47.33) | 15(2.76) | 2(0.37) |
| 8. I believe that I can adjust the dosage or interval of biologic therapies by myself to increase efficacy or when symptoms worsen. N | 13(2.39) | 66(12.15) | 275(50.64) | 149(27.44) | 40(7.37) |
| 9. I believe that using biologic therapies is a long-term commitment in the treatment of systemic lupus erythematosus. P | 37(6.81) | 181(33.33) | 305(56.17) | 19(3.50) | 1(0.18) |
| 10. I am willing to try new biologic therapies if they become available. P | 35(6.45) | 132(24.31) | 337(62.06) | 34(6.26) | 5(0.92) |
| 11. How willing are you to use the following biologic therapies? (All to be answered) |  |  |  |  |  |
| Belimumab | 54(9.94) | 114(20.99) | 339(62.43) | 27(4.97) | 9(1.66) |
| Rituximab | 8(1.47) | 45(8.29) | 429(79.01) | 35(6.45) | 26(4.79) |
| Tocilizumab | 17(3.13) | 66(12.15) | 405(74.59) | 34(6.26) | 21(3.87) |
| Tofacitinib | 7(1.29) | 41(7.55) | 436(80.29) | 33(6.08) | 26(4.79) |
| JAK inhibitors | 9(1.66) | 39(7.18) | 436(80.29) | 34(6.26) | 25(4.60) |
| Tumor necrosis factor antagonists | 8(1.47) | 36(6.63) | 437(80.48) | 34(6.26) | 28(5.16) |
| Low-dose IL-2 | 8(1.47) | 38(7.00) | 438(80.66) | 32(5.89) | 27(4.97) |

**Supplementary table 3 Responses for Each Item in the Practice Dimension**

| Items, n (%) | Very willing | Willing | Neutral | Unwilling | Very unwilling |
| --- | --- | --- | --- | --- | --- |
| 1. If biologics is needed, I will strictly follow the doctor's instructions for using biologic therapies. | 142(26.15) | 257(47.33) | 139(25.60) | 5(0.92) | 0 |
| 1. If biologics is needed, I will closely monitor the progression or changes of my own disease. | 148(27.26) | 263(48.43) | 126(23.20) | 4(0.74) | 2(0.37) |
| 3. During the use of biologic therapies, I am willing to closely monitor for adverse reactions to the medication. | 148(27.26) | 274(50.46) | 117(21.55) | 2(0.37) | 2(0.37) |
| 4. I am willing to undergo regular check-ups during medication use to understand the progression of the disease and the effectiveness of the medication. | 156(28.72) | 274(50.46) | 111(20.44) | 0 | 2(0.37) |
| 5. I am willing to recommend biologic therapies that have been effective for me to other patients. | 111(20.44) | 245(45.12) | 179(32.97) | 6(1.10) | 2(0.37) |
| 7. I am willing to actively learn about the knowledge and latest developments of biologic therapies. | 137(25.23) | 269(49.54) | 134(24.68) | 1(0.18) | 2(0.37) |

**Supplementary table 4 SEM Model Effectiveness**

| Indicators | Reference | Actual |
| --- | --- | --- |
| CMIN/DF | 1-3: Excellent, 3-5: Good | 2.727 |
| RMSEA | <0.08: Good | 0.056 |
| IFI | >0.8: Good | 0.945 |
| TLI | >0.8: Good | 0.937 |
| CFI | >0.8: Good | 0.945 |
